# Supplementary material for: Gut Microbiota Associated with Gestational Health Conditions in a Sample of Mexican Women
Source: Nutrients. 2022 Nov 14;14(22):4818. doi: 10.3390/nu14224818 (PMC9696207; doi:10.3390/nu14224818)
Supplement: Supplementary file 1 [file nutrients-14-04818-s001.zip › nutrients-2001080-supplementary.pdf]

nutrients

Article

Gut microbiota associated with gestational health conditions in a sample of Mexican Women.

Tizziani Benitez-Guerrero [1,†], Juan Manuel Vélez-Ixta [1,†], Carmen Josefina Juárez-Castelán [1], Karina Corona-Cervantes [1], Alberto Piña-Escobedo [1], Helga Martínez-Corona [1], Amapola De Sales-Millán [1,$], Yair Cruz-Narváez [2], Carlos Yamel Gómez-Cruz [2], Tito Ramirez-Lozada [3], Gustavo Acosta-Altamirano [4], Mónica Sierra-Martínez [5], Paola Berenice Zárate-Segura [6], and Jaime García-Mena [1,*]

[1] Departamento de Genética y Biología Molecular, Cinvestav, Av. Instituto Politécnico Nacional 2508, Ciudad de México, 07360, Mexico

[2] Laboratorio de Posgrado de Operaciones Unitarias, Escuela Superior de Ingeniería Química e Industrias Extractivas, Instituto Politécnico Nacional, Ciudad de México, 07738, Mexico

[3] Unidad de Ginecología y Obstetricia. Hospital Regional de Alta Especialidad de Ixtapaluca. Carretera Federal México-Puebla Km. 34.5, Col. Zoquiapan, Ixtapaluca, Estado de México, 56530, Mexico

[4] Dirección de Planeación, Enseñanza e Investigación. Hospital Regional de Alta Especialidad de Ixtapaluca. Carretera Federal México-Puebla Km. 34.5, Col. Zoquiapan, Ixtapaluca, Estado de México, 56530, Mexico

[5] Unidad de Investigación en Salud. Hospital Regional de Alta Especialidad de Ixtapaluca. Carretera Federal México-Puebla Km. 34.5, Col. Zoquiapan, Ixtapaluca, Estado de México, 56530, Mexico

[6] Laboratorio de Medicina Traslacional, Escuela Superior de Medicina, Instituto Politécnico Nacional, Ciudad de México, 11340, Mexico

* Correspondence: jgmena@cinvestav.mx (J.G.-M.);
Tel.: +52-(55)−5747−3800 (ext. 5327) (J.G.-M.)

† These authors contributed equally to this work.

$ Current Address: Universidad Autónoma Metropolitana, Unidad Lerma. Doctorado en Ciencias Biológicas y de la Salud.

Supplementary Material.

Tables.

Table S1. Macronutrients with statistical significance between groups

Group 1	Group 2	Macronutrient	*p*-value
CO	GD	Energy Intake (kcal/day)	0.0033**
CO	GD	Carbohydrates intake (g/day)	0.0470*
CO	GD	Protein intake (g/day)	0.0200*
CO	GD	Total fiber intake (g/day)	0.0160*
CO	PE	Starch (g/day)	0.0520
CO	GD	Cereal (kcal/day)	0.0039**
CO	GD	Sucrose (g/day)	0.0730
CO	GD	Fructose (g/day)	0.1100
CO	GD	Glucose (g/day)	0.1000
CO	PE	Glucose (g/day)	0.1000
CO	GD	Sodium (mg/day)	0.0024**

CO, Controls; GD, Gestational Diabetes; PD, Pregestational Diabetes; PE, Pre-Eclampsia. The *p*-value was calculated based on Pairwise Wilcoxon test, performing a Benjamini- Hochberg *post-hoc* test. *$p \leq 0.05$; **$p \leq 0.01$.

Table S2. Alpha-diversity indices of the study groups

Index	CO	GD	PD	PE	*p*-value		
					CO/DG	CO/PD	CO/PE
Chao1	120.10 ± 65.17	116.46 ± 69.41	117.55 ± 54.97	110.67 ± 42.95	0.85	0.73	1.00
ACE	121.96 ± 64.74	113.42 ± 65.93	119.64 ± 56.45	111.14 ± 43.14	0.61	0.77	0.87
Shannon	2.47 ± 0.95	2.13 ± 0.96	2.73 ± 0.34	2.53 ± 0.91	0.36	0.73	0.82
Simpson	0.74 ± 0.22	0.67 ± 0.28	0.88 ± 0.04	0.75 ± 0.25	0.41	0.39	0.79
InvSimpson	7.94 ± 6.56	5.61 ± 3.99	8.75 ± 2.62	8.14 ± 5.65	0.41	0.39	0.79
Fisher	20.03 ± 13.07	17.21 ± 10.66	19.78 ± 10.30	18.64 ± 8.56	0.65	0.76	0.84

CO, Control; GD; Gestational diabetes; PD, Pre-gestational diabetes; PE, Preeclampsia. The *p*-value was calculated according to U-Mann Whitney test.; $p < 0.05$ are considered statistically significant differences. The data shown is the mean ± standard deviation.

Table S3. Relative abundance at phylum level in Control versus Gestational Diabetes groups

Phylum	Group	%	CV	Range	*p*-value
Proteobacteria	CO	62.77	33.45	0.93-97.60	0.2188
	GD	46.82	43.21	0.08-96.81	
Actinobacteriota	CO	12.22	9.13	0.04-36.37	0.7139
	GD	14.35	28.33	0.73-98.92	
Bacteroidota	CO	3.38	11.13	0.03-61.05	0.4007
	GD	0.00	0.00	0.00-0.00	
Firmicutes	CO	21.15	29.58	0.45-98.22	0.1629
	GD	38.02	43.37	0.54-98.01	
Other	CO	0.35	0.43	0.00-1.57	0.0813
	GD	0.66	0.63	0.06-1.99	

CO, Control (n = 30); GD, Gestational Diabetes (n = 11); %, Relative abundance of each taxon; SD, Standard Deviation; CV, Coefficient of Variation; Range, minimum and maximum values in the relative abundance for each taxon. The *p*-value was calculated according to T-test. $p < 0.05$ are considered statistically significant differences.

Table S4. Relative abundance at phylum level in Control versus Pre-gestational Diabetes groups

Phylum	Group	%	CV	Range	*p*-value
Proteobacteria	CO	62.77	33.45	0.93-97.60	0.2899
	PD	79.08	3.15	75.53-83.48	
Actinobacteriota	CO	12.22	9.13	0.04-36.37	0.2716
	PD	16.86	1.86	15.00-19.44	
Bacteroidota	CO	3.38	11.13	0.03-61.05	0.4007
	PD	0.00	0.00	0.00-0.00	
Firmicutes	CO	21.15	29.58	0.45-98.22	0.1771
	PD	2.67	1.76	0.43-4.69	
Other	CO	0.35	0.43	0.00-1.57	0.0005***
	PD	1.32	0.94	0.75-2.97	

CO, Control (n = 30); PD, Pre-gestational Diabetes (n = 5); %, Relative abundance of each taxon; SD, Standard Deviation; CV, Coefficient of Variation; Range, minimum and maximum values in the relative abundance for each taxon. The *p*-value was calculated according to T-test; ***$p \leq 0.001$ are considered statistically significant differences.

Table S5. Relative abundance at phylum level in Control versus Pre-eclampsia groups

Phylum	Group	%	CV	Range	*p*-value
Proteobacteria	CO	62.77	33.45	0.93-97.60	0.7699
	PE	58.92	30.48	1.12-87.89	
Bacteroidota	CO	3.38	11.13	0.03-61.05	0.5682
	PE	1.09	1.20	0.22-3.87	
Actinobacteriota	CO	12.22	9.13	0.04-36.37	0.7918
	PE	13.19	9.40	0.97-25.77	
Firmicutes	CO	21.15	29.58	0.45-98.22	0.7013
	PE	25.82	33.46	2.42-96.28	
Other	CO	0.35	0.43	0.00-1.57	0.0362*
	PE	0.71	0.35	0.35-1.42	

CO, Control (n = 30); PE, Preeclampsia (n = 8); %, Relative abundance of each taxon; SD, Standard Deviation; CV, Coefficient of Variation; Range, minimum and maximum values in the relative abundance for each taxon. The *p*-value was calculated according to T-test; *$p \leq 0.05$ are considered statistically significant differences.

Table S6. Relative abundance at genus level in Control versus Gestational Diabetes groups

Phylum	Genus	Group	% ± SD	CV	Range	*p*-value
Proteobacteria	*Achromobacter*	CO	3.01	3.82	0.01-16.33	<0.0001***
		GD	0.00	0.00	0.00-0.00	
Proteobacteria	*Allorhizobium-Neorhizobium-Pararhizobium-Rhizobium*	CO	1.28	1.49	0.02-6.72	0.0002***
		GD	0.00	0.00	0.00-0.00	
Bacteroidota	*Bacteroides*	CO	2.98	11.75	0.01-57.78	0.2207
		GD	0.00	0.00	0.00-0.00	
Actinobacteriota	*Bifidobacterium*	CO	1.67	4.15	0.01-20.91	0.0378*
		GD	0.00	0.00	0.00-0.00	
Firmicutes	*Blautia*	CO	4.29	6.52	0.01-20.51	0.1010
		GD	17.27	16.01	0.13-35.68	
Proteobacteria	*Bosea*	CO	4.47	5.75	0.02-23.41	0.2225
		GD	11.00	21.30	0.00-53.26	
Proteobacteria	*Bradyrhizobium*	CO	3.56	2.90	0.07-9.50	0.7606
		GD	4.98	6.40	0.01-14.97	
Firmicutes	*Clostridium_sensu_stricto_1*	CO	8.85	23.10	0.00-89.67	0.9734
		GD	9.38	22.71	0.01-72.32	
Actinobacteriota	*Cutibacterium*	CO	2.78	2.75	0.01-10.90	0.0122*
		GD	0.00	0.00	0.00-0.00	
Proteobacteria	*Enterobacter*	CO	0.00	0.00	0.00-0.00	0.0731
		GD	4.17	6.42	0.01-15.57	
Firmicutes	*Enterococcus*	CO	2.08	4.24	0.01-15.08	0.0977
		GD	5.78	11.69	0.02-31.11	
Proteobacteria	*Escherichia-Shigella*	CO	3.26	4.98	0.02-22.53	0.2592
		GD	8.35	24.03	0.04-80.61	
Proteobacteria	*Mesorhizobium*	CO	2.39	1.72	0.04-7.40	0.0156*
		GD	0.00	0.00	0.00-0.00	
Proteobacteria	*Methylobacterium-Methylorubrum*	CO	0.00	0.00	0.00-0.00	0.0562
		GD	2.19	2.24	0.08-5.68	
Actinobacteriota	*Microbacterium*	CO	5.21	4.33	0.02-15.94	0.0655
		GD	2.96	3.05	0.03-7.86	
Firmicutes	*Paraclostridium*	CO	1.99	3.25	0.01-10.53	0.2113
		GD	7.15	10.89	0.07-24.86	
Proteobacteria	*Pseudomonas*	CO	2.36	2.78	0.02-13.02	0.0865
		GD	0.00	0.00	0.00-0.00	
Actinobacteriota	*Rothia*	CO	0.00	0.00	0.00-0.00	0.2862
		GD	17.16	40.05	0.03-98.85	
Proteobacteria	*Sphingomonas*	CO	12.32	11.18	0.02-39.34	0.0537
		GD	4.36	6.24	0.00-19.20	
Firmicutes	*Staphylococcus*	CO	0.00	0.00	0.00-0.00	0.1979
		GD	2.86	6.77	0.03-21.93	
Firmicutes	*Streptococcus*	CO	0.00	0.00	0.00-0.00	0.0690
		GD	2.01	3.26	0.05-9.07	
----	Other	CO	15.80	16.46	0.13-59.78	0.7705
		GD	14.22	10.98	0.32-37.90	

CO, Control (n = 30); GD, Gestational Diabetes (n = 11); %, Relative abundance of each taxon; SD, Standard Deviation; CV, Coefficient of Variation; Range, minimum and maximum values in the relative abundance for each taxon. The *p*-value was calculated according to T-test; *$p < 0.05$, ***$p < 0.001$ are considered statistically significant differences.

Table S7. Relative abundance at genus level in Control versus Pre-gestational Diabetes groups

Phylum	Genus	Group	% ± SD	CV	Range	*p*-value
Proteobacteria	*Achromobacter*	CO	3.01	3.82	0.01-16.33	0.5497
		PD	4.18	8.99	0.05-20.26	
Proteobacteria	*Allorhizobium-Neorhizobium-Pararhizobium-Rhizobium*	CO	1.28	1.49	0.02-6.72	0.0005***
		PD	5.24	1.97	3.58-7.56	
Bacteroidota	*Bacteroides*	CO	2.98	11.75	0.01-57.78	0.2207
		PD	0.00	0.00	0.00-0.00	
Actinobacteriota	*Bifidobacterium*	CO	1.67	4.15	0.01-20.91	0.0378*
		PD	0.00	0.00	0.00-0.00	
Firmicutes	*Blautia*	CO	4.29	6.52	0.01-20.51	0.2030
		PD	0.00	0.00	0.00-0.00	
Proteobacteria	*Bosea*	CO	4.47	5.75	0.02-23.41	0.9009
		PD	4.80	3.10	0.96-7.88	
Proteobacteria	*Bradyrhizobium*	CO	3.56	2.90	0.07-9.50	0.7357
		PD	4.61	2.35	3.17-8.11	
Firmicutes	*Clostridium_sensu_stricto_1*	CO	8.85	23.10	0.00-89.67	0.2321
		PD	0.00	0.00	0.00-0.00	
Actinobacteriota	*Cutibacterium*	CO	2.78	2.75	0.01-10.90	0.0503
		PD	5.52	4.34	1.76-10.24	
Firmicutes	*Enterococcus*	CO	2.08	4.24	0.01-15.08	0.2855
		PD	0.00	0.00	0.00-0.00	
Proteobacteria	*Escherichia-Shigella*	CO	3.26	4.98	0.02-22.53	0.4607
		PD	1.44	2.98	0.04-6.78	
Proteobacteria	*Mesorhizobium*	CO	2.39	1.72	0.04-7.40	0.6474
		PD	3.09	1.33	1.16-4.10	
Proteobacteria	*Methylobacterium-Methylorubrum*	CO	0.00	0.00	0.00-0.00	0.0201*
		PD	1.28	1.70	0.22-4.23	
Actinobacteriota	*Microbacterium*	CO	5.21	4.33	0.02-15.94	0.1889
		PD	7.83	5.82	1.30-12.90	
Firmicutes	*Paraclostridium*	CO	1.99	3.25	0.01-10.53	0.1505
		PD	0.00	0.00	0.00-0.00	
Proteobacteria	*Pseudomonas*	CO	2.36	2.78	0.02-13.02	<0.0001***
		PD	14.07	8.91	0.33-22.04	
Proteobacteria	*Sphingomonas*	CO	12.32	11.18	0.02-39.34	0.0653
		PD	21.35	5.36	16.09-30.06	
Proteobacteria	*Sphingopyxis*	CO	0.00	0.00	0.00-0.00	0.0564
		PD	11.06	8.51	1.10-21.87	
Proteobacteria	*Undibacterium*	CO	0.00	0.00	0.00-0.00	0.3436
		PD	8.24	16.37	0.02-32.79	
-----	Other	CO	15.80	16.46	0.13-59.78	0.5397
		PD	11.17	3.09	6.74-14.26	

CO, Control (n = 30); PD, Pre-gestational Diabetes (n = 5); %, Relative abundance of each taxon; SD, Standard Deviation; CV, Coefficient of Variation; Range, minimum and maximum values in the relative abundance for each taxon. The *p*-value was calculated according to T-test; $*p < 0.05$, and $***p < 0.001$ are considered statistically significant differences.

Table S8. Relative abundance at genus level in Control versus Pre-eclampsia groups

Phylum	Genus	Group	% ± SD	CV	Range	*p*-value
Proteobacteria	*Achromobacter*	CO	3.01	3.82	0.01-16.33	0.6066
		PE	6.15	10.27	0.02-23.77	
Proteobacteria	*Allorhizobium-Neorhizobium-Pararhizobium-Rhizobium*	CO	1.28	1.49	0.02-6.72	0.1324
		PE	2.58	3.44	0.13-9.75	
Bacteroidota	*Bacteroides*	CO	2.98	11.75	0.01-57.78	0.2207
		PE	0.00	0.00	0.00-0.00	
Actinobacteriota	*Bifidobacterium*	CO	1.67	4.15	0.01-20.91	0.7945
		PE	2.00	4.68	0.08-13.56	
Firmicutes	*Blautia*	CO	4.29	6.52	0.01-20.51	0.2030
		PE	0.00	0.00	0.00-0.00	
Proteobacteria	*Bosea*	CO	4.47	5.75	0.02-23.41	0.0525
		PE	10.08	12.23	0.00-29.99	
Proteobacteria	*Bradyrhizobium*	CO	3.56	2.90	0.07-9.50	0.1161
		PE	5.98	3.63	1.33-11.14	
Firmicutes	*Clostridium_sensu_stricto_1*	CO	8.85	23.10	0.00-89.67	0.2321
		PE	0.00	0.00	0.00-0.00	
Actinobacteriota	*Corynebacterium*	CO	0.00	0.00	0.00-0.00	0.0012**
		PE	1.30	0.70	0.46-2.57	
Actinobacteriota	*Cutibacterium*	CO	2.78	2.75	0.01-10.90	0.1739
		PE	4.71	2.51	1.49-8.75	
Proteobacteria	*Enterobacter*	CO	0.00	0.00	0.00-0.00	0.0546
		PE	1.40	2.08	0.00-5.68	
Firmicutes	*Enterococcus*	CO	2.08	4.24	0.01-15.08	0.0865
		PE	14.99	35.79	0.02-88.05	
Proteobacteria	*Escherichia-Shigella*	CO	3.26	4.98	0.02-22.53	0.1507
		PE	10.84	23.88	0.14-64.42	
Firmicutes	*Finegoldia*	CO	0.00	0.00	0.00-0.00	0.1030
		PE	1.33	1.98	0.10-5.71	
Firmicutes	*Gemella*	CO	0.00	0.00	0.00-0.00	0.0904
		PE	1.59	2.26	0.11-6.11	
Proteobacteria	*Mesorhizobium*	CO	2.39	1.72	0.04-7.40	0.0156*
		PE	0.00	0.00	0.00-0.00	
Proteobacteria	*Methylobacterium-Methylorubrum*	CO	0.00	0.00	0.00-0.00	0.0025**
		PE	2.28	1.85	0.63-5.82	
Actinobacteriota	*Microbacterium*	CO	5.21	4.33	0.02-15.94	0.3951
		PE	3.91	3.37	0.46-10.69	
Firmicutes	*Paraclostridium*	CO	1.99	3.25	0.01-10.53	0.1505
		PE	0.00	0.00	0.00-0.00	
Proteobacteria	*Pseudomonas*	CO	2.36	2.78	0.02-13.02	0.5974
		PE	3.30	4.95	0.12-13.39	
Proteobacteria	*Sphingomonas*	CO	12.32	11.18	0.02-39.34	0.4124
		PE	9.22	5.11	3.48-19.33	
Firmicutes	*Staphylococcus*	CO	0.00	0.00	0.00-0.00	0.0516
		PE	6.47	10.37	0.01-29.02	
Firmicutes	*Streptococcus*	CO	0.00	0.00	0.00-0.00	0.0306*
		PE	2.64	3.75	0.31-10.43	
	Other	CO	15.80	16.46	0.13-59.78	0.5398
		PE	12.10	5.86	4.53-19.62	

CO, Control (n = 30); PE, Preeclampsia (n = 8); %, Relative abundance of each taxon; SD, Standard Deviation; CV, Coefficient of Variation; Range, minimum and maximum values in the relative abundance for each taxon. The *p*-value was calculated according to T-test; $*p < 0.05$, $**p < 0.01$ are considered statistically significant differences.

Table S9. DESeq2 comparative analysis for Control versus Gestational Diabetes groups

Group	Phylum	Genus	log2FoldChange	*p*-value	*p*-adjusted value
CO	Firmicutes	*Hungatella*	-23.448718	2.06E-14	4.37E-12
CO	Firmicutes	*Clostridium_sensu_stricto_1*#	-24.188217	2.91E-13	2.32E-11
CO	Firmicutes	*Clostridium_sensu_stricto_1*#	-23.766956	7.46E-13	4.76E-11
CO	Bacteroidota	*Bacteroides*$	-23.657222	9.50E-13	5.05E-11
CO	Bacteroidota	*Bacteroides*$	-22.263813	1.89E-11	6.70E-10
CO	Cyanobacteria	*Obscuribacteraceae*	-5.995397	0.000825	0.023923
CO	Firmicutes	*Clostridium_sensu_stricto_1*#	-3.674582	0.001652	0.043913
CO	Proteobacteria	*Pseudomonas*	-7.427341	0.001815	0.044534
GD	Firmicutes	*Staphylococcus*	24.946798	2.74E-14	4.37E-12
GD	Firmicutes	*Clostridium_sensu_stricto_1*#	24.291440	1.26E-13	1.34E-11
GD	Firmicutes	*Clostridia_UCG-014*	22.593262	5.78E-12	2.63E-10
GD	Proteobacteria	*Enterobacter*	22.066789	1.81E-11	6.70E-10
GD	Actinobacteriota	*Rothia*	9.864361	0.000179	0.005705

CO, Control; GD, Gestational diabetes; the *p*-value was calculated according to Wald test, differences were considered significant with the *p*-value (adjusted for false discovery rate using Benjamini–Hochberg correction) was of 0.05. #, four different ASVs were reported for *Clostridium_sensu_stricto_1* in the analysis. $, two different ASVs were reported for *Bacteroides* in the analysis.

Table S10. DESeq2 comparative analysis for Control versus Pre-gestational Diabetes groups

Group	Phylum	Genus	log2FoldChange	p-value	p-adjusted value
CO	Firmicutes	Clostridium_sensu_stricto_1#	-24.287390	8.01E-25	1.03E-22
CO	Firmicutes	Blautia	-23.518789	8.88E-11	3.21E-09
CO	Firmicutes	Lachnoclostridium	-21.317621	8.56E-10	2.21E-08
CO	Firmicutes	Clostridium_sensu_stricto_1#	-22.583852	8.42E-09	1.81E-07
CO	Firmicutes	Fusicatenibacter	-22.755033	7.10E-08	9.46E-07
CO	Firmicutes	Hungatella	-21.357933	6.90E-08	9.46E-07
CO	Firmicutes	Clostridium_sensu_stricto_1#	-22.639536	8.27E-08	9.70E-07
CO	Firmicutes	UCG-002	-22.332805	1.23E-07	1.22E-06
CO	Firmicutes	Clostridium_sensu_stricto_1#	-21.989757	1.92E-07	1.65E-06
CO	Firmicutes	Lachnospiraceae_NK4A136_group	-22.040660	1.80E-07	1.65E-06
CO	Firmicutes	Dorea	-21.855516	2.28E-07	1.82E-06
CO	Actinobacteriota	Bifidobacterium	-21.814470	2.40E-07	1.82E-06
CO	Firmicutes	Faecalibacterium	-21.410804	3.99E-07	2.75E-06
CO	Bacteroidota	Bacteroides$	-21.396541	4.06E-07	2.75E-06
CO	Firmicutes	UBA1819	-21.053919	6.20E-07	4.00E-06
CO	Firmicutes	uncultured	-20.618324	1.05E-06	6.18E-06
CO	Bacteroidota	Bacteroides$	-20.261214	1.62E-06	9.08E-06
CO	Firmicutes	Enterococcus	-7.1399532	0.002964	0.015291
PD	Proteobacteria	Pseudomonas	2.7364358	0.007854	0.038969

CO, Control; PD, Pre-gestational diabetes; the p-value was calculated according to Wald test, differences were considered significant with the p-value (adjusted for false discovery rate using Benjamini–Hochberg correction) was of 0.05. #, four different ASVs were reported for Clostridium_sensu_stricto_1 in the analysis. $, two different ASVs were reported for Bacteroides in the analysis.

Table S11. DESeq2 comparative analysis for Control versus Pre-Eclampsia groups

Group	Phylum	Genus	log2FoldChange	*p*-value	*p*-adjusted value
CO	Firmicutes	*Blautia*	-25.906744	7.32E-13	6.04E-11
CO	Firmicutes	*Faecalibacterium*	-24.536049	5.18E-13	6.04E-11
CO	Firmicutes	*Hungatella*	-22.326538	2.39E-11	9.85E-10
CO	Firmicutes	*Clostridium_sensu_stricto_1*[#]	-23.538588	7.18E-11	1.97E-09
CO	Firmicutes	*UCG-002*	-23.292967	1.13E-10	2.66E-09
CO	Firmicutes	*Clostridium_sensu_stricto_1*[#]	-22.885432	2.36E-10	3.90E-09
CO	Firmicutes	*Dorea*	-22.931249	2.18E-10	3.90E-09
CO	Firmicutes	*Lachnospiraceae_NK4A136_group*	-22.995676	1.94E-10	3.90E-09
CO	Firmicutes	*[Eubacterium]_hallii_group*	-22.609963	3.87E-10	5.81E-09
CO	Bacteroidota	*Bacteroides*[$]	-22.543879	4.35E-10	5.99E-09
CO	Firmicutes	uncultured	-21.572836	2.37E-09	2.79E-08
CO	Bacteroidota	*Bacteroides*[$]	-21.219866	4.31E-09	4.74E-08
CO	Firmicutes	*Clostridium_sensu_stricto_1*[#]	-6.7004416	1.63E-06	1.68E-05
CO	Actinobacteriota	*Iamia*	-7.442506	2.01E-06	1.95E-05
CO	Actinobacteriota	*Mycobacterium*	-7.386467	5.39E-06	4.94E-05
CO	Firmicutes	*Paraclostridium*	-7.5188684	0.000117	0.001012
CO	Cyanobacteria	*Obscuribacteraceae*	-5.750866	0.003494	0.027450
PE	Firmicutes	*Staphylococcus*	2.735962	0.000983	0.008110
PE	Firmicutes	*Gemella*	3.725659	0.003870	0.029023

CO, Control; PE, Preeclampsia; the *p*-value was calculated according to Wald test, differences were considered significant with the *p*-value (adjusted for false discovery rate using Benjamini–Hochberg correction) was of 0.05. #, three different ASVs were reported for *Clostridium_sensu_stricto_1* in the analysis. $, two different ASVs were reported for *Bacteroides* in the analysis.

Table S12. Comparative differences between means, *p*, and corrected *p*-values for the predicted metagenomes

Group	Feature	Differences between means	*p*-value	Corrected *p*-value
CO/GD	Ectoine biosynthesis	0.0378	0.0001	0.0451
	Meta cleavage pathway of aromatic compounds	0.0181	$3.94e^{-5}$	0.0165
	Vitamin B6 degradation	0.0069	$1.82e^{-6}$	0.0008
	2-nitrobenzoate degradation I	0.0183	$1.68e^{-5}$	0.0070
	2-amino-3-carboxymuconate semialdehyde degradation to 2-Oxopentenoate	0.0180	$4.58e^{-5}$	0.0191
	L-tryptophan degradation IX	0.0196	$3.52e^{-5}$	0.0147
	2-aminophenol degradation	0.0081	$9.06e^{-5}$	0.0379
	L-tryptophan degradation XII (Geobacillus)	0.0195	$4.07e^{-5}$	0.0170
	Superpathway of sulfolactate degradation	0.0196	0.0001	0.0453
	Methylaspartate cycle	0.0282	$9.94e^{-6}$	0.0042
	Androstenedione degradation	0.0037	$4.24e^{-5}$	0.0177
	1,5-anhydrofructose degradation	0.0301	$1.78e^{-7}$	$7.43e^{-5}$
	Nicotinate degradation I	0.0189	$1.33e^{-5}$	0.0056
CO/PD	Adenosylcobalamin salvage from cobinamide I	0.1167	$3.00e^{-6}$	0.0013
	Colanic acid building blocks biosynthesis	-0.1269	$2.45e^{-5}$	0.0102
	D-galacturonate degradation I	0.1024	$8.14e^{-5}$	0.0340
	Superpathway of glycolysis, pyruvate dehydrogenase, TCA, and glyoxylate bypass	-0.1400	$5.51e^{-6}$	0.0023
	L-methionine biosynthesis I	0.1636	$1.68e^{-6}$	0.0007
	L-leucine degradation I	-0.2015	$2.60e^{-6}$	0.0011
	Superpathway of S-adenosyl-L-methionine biosynthesis	0.1133	$1.42e^{-6}$	0.0006
	Superpathway of L-lysine, L-threonine and L-methionine biosynthesis I	0.0849	0.0001	0.0474
	Myo-inositol degradation I	0.1699	$4.40e^{-5}$	0.0184
	Superpathway of L-methionine biosynthesis (transsulfuration)	0.1325	$1.88e^{-7}$	$7.88e^{-5}$
	Adenosylcobalamin biosynthesis from cobyrinate a,c-diamide I	0.1111	$5.59e^{-5}$	0.0234
	GDP-mannose biosynthesis	-0.1655	$2.29e^{-6}$	0.0010
	TCA cycle VI (obligate autotrophs)	-0.1571	$1.12e^{-5}$	0.0047
	Superpathay of heme biosynthesis from glutamate	-0.1209	$8.15e^{-6}$	0.0034
	Purine nucleotides degradation II (aerobic)	0.1704	$2.85e^{-8}$	$1.19e^{-5}$
	Guanosine nucleotides degradation III	0.1753	$1.91e^{-5}$	0.0080
	Adenine and adenosine salvage III	0.2275	$6.62e^{-5}$	0.0277
	Superpathway of L-phenylalanine biosynthesis	-0.1161	$7.93e^{-7}$	0.0003
	Superpathway of L-tyrosine biosynthesis	-0.1074	$1.13e^{-6}$	0.0005
	Myo-, chiro- and scillo-inositol degradation	0.3128	$2.51e^{-5}$	0.0105
	Superpathway of GDP-mannose-derived O-antigen building blocks biosynthesis	-0.1253	$4.10e^{-5}$	0.0171
	Superpathway of UDP-glucose-derived O-antigen building blocks biosynthesis	-0.1319	$1.33e^{-5}$	0.0056
	Purine ribonucleosides degradation	0.2452	$1.36e^{-6}$	0.0006
	Superpathway of purine deoxyribonucleosides degradation	0.1593	$1.26e^{-5}$	0.0053
	TCA cycle VIII (helicobacter)	-0.1999	$1.35e^{-5}$	0.0057
	L-tyrosine degradation I	-0.1491	$4.22e^{-7}$	0.0002
	Superpathway of ubiquinol-8 biosynthesis (prokaryotic)	-0.1362	$1.80e^{-6}$	0.0008
CO/PE	Vitamin B6 degradation	0.0060	$6.46e^{-5}$	0.0270

CO, control; PD, pre-gestational diabetes; PE, preeclampsia. Welch's test was applied with a Bonferroni pos-hoc. $p < 0.01$ was considered statistically significant.

Table S13. Metabolites identified with positive ionization with ESI FT-ICR mass spectral analysis

Compound name	Candidate structure	m/z$^{\$}$	m/z$^{\&}$	Error	ID	Group	Intensity ratio
trioxopyrrolopyridine	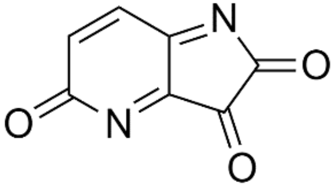	145.00296	145.00325	2.05	25937555	CO	2.2866
						GD	2.6453
						PD	1.7453
						PE	2.0165
9,9'-spirobi[carbazol-9-ium]	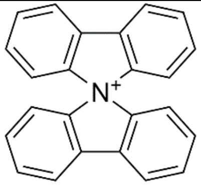	318.12683	318.12773	2.81	77408521	CO	2.0124
						GD	1.0300
(2S)-2-amino-5-[2-[2-[2-[2-[2-[2-[(5,6-diamino-6-keto-hexyl)amino]-2-keto-ethoxy]ethoxy]ethylamino]-2-keto-ethoxy]ethoxy]ethylamino]-5-keto-valeric acid	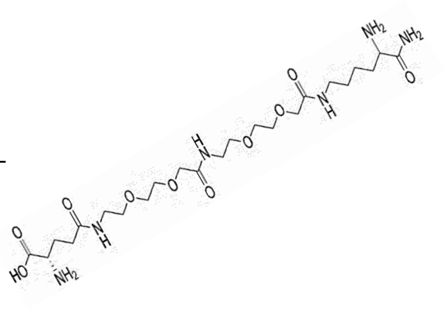	603.27465	603.27505	0.66	CID 144253942	CO	1.0000
1-[4-[3,5-bis[4-(2,4,4,5,5-pentamethyl-2-imidazolin-1-yl)phenyl]phenyl]phenyl]-2,4,4,5,5-pentamethyl-2-imidazoline	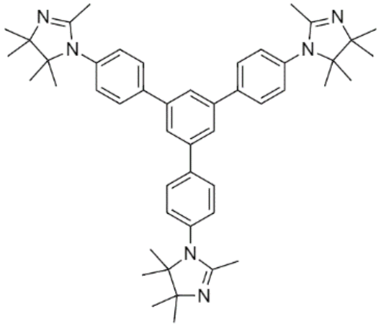	720.48751	720.48740	-0.16	CID 135237206	CO	2.3488
						GD	1.8389
						PD	2.0391
						PE	2.0688
2-acetamido-5-[[4-[[1-carboxy-4-keto-4-[[4-keto-4-[[6-keto-5-(propylamino)undecyl]amino]-1-methyl-butyl]amino]butyl]amino]-4-keto-1-methyl-butyl]amino]-5-keto-valeric acid		779.43169	779.43155	-0.18	CID 124033363	CO	6.7533

	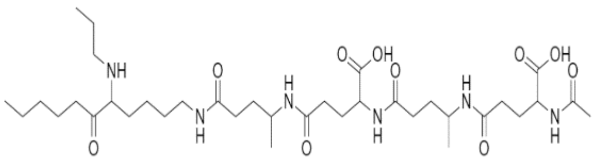					GD	4.2477
						PD	9.4718
						PE	2.9899
5-[2-[2-[2-[2-(2-acetonyloxyethoxy)ethylamino]-2-keto-ethoxy]ethoxy]ethylamino]-5-keto-2-[16-(2H-tetrazol-5-yl)hexadecanoylamino]valeric acid	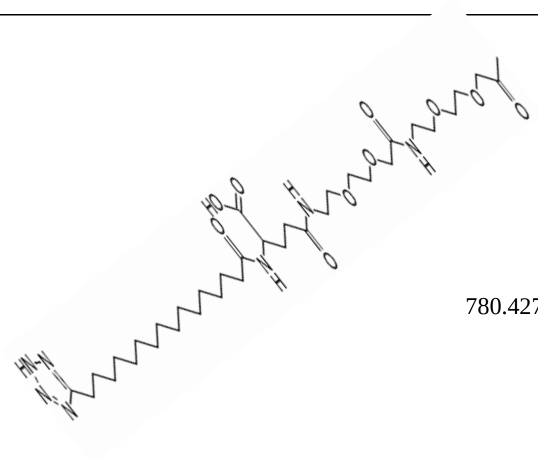	780.42731	780.42680	-0.66	CID 77486865	CO	1.6344
						GD	1.3474
						PD	1.4480
						PE	1.9591
20-[[(1S)-1-[[(1S)-1-[2-(2-acetonyloxyethoxy)ethylcarbamoyl]-3-carboxy-propyl]carbamoyl]-3-carboxy-propyl]amino]-20-keto-arachidic acid	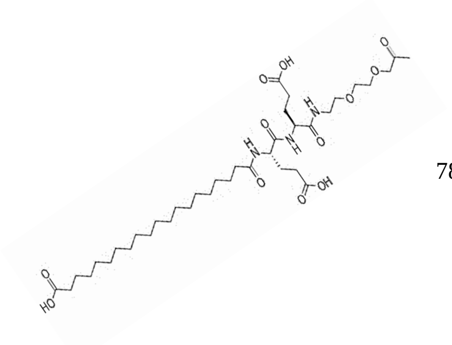	782.42057	782.41998	-0.75	CID 143776451	CO	1.6760
						GD	1.5693
						PD	2.2254

1,1,1,21,21,21-Hexaphenyl-2,5,8,11,14,17,20-heptaoxahenicosane	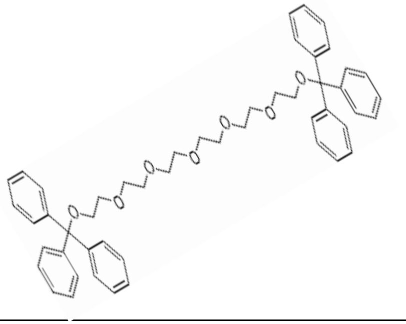	784.42075	784.42078	0.03	8638281	CO	1.5389
						GD	1.0797
						PD	2.0758
						PE	1.9452
9-(4-butylphenyl)-14-[3-ethyl-3-[2-(5-phenyl-2-propyl-1,2,4-triazol-3-yl)pyridin-1-ium-1-yl]cyclobutyl]-22-methyl-9-aza-15-azoniahexacyclo[11.11.0.02,10.03,8.015,24.016,21]tetracosa-1(13),2(10),3,5,7,11,15(24),16,18,20,22-undecaene	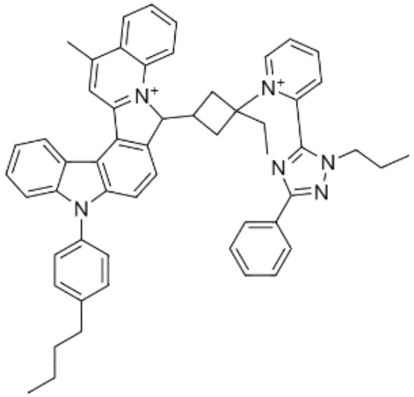	837.40347	837.40415	0.82	CID 123394094	CO	2.9450
						GD	2.0372
						PD	2.6721
						PE	2.4735
10-keto-10-[3-[10,15,20-tris(p-tolyl)-21,23-dihydroporphin-5-yl]phenoxy]capric acid	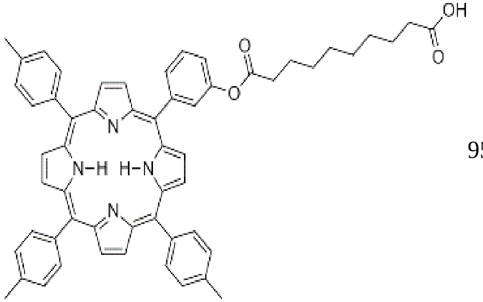	95.36211	895.36201	-0.10	CID 135926152	CO	1.0000

[4-[3-[4-[[4-[[4-[3-(4-aminophenyl)-5-(p-tolyl)phenyl]phenyl]iminomethyl]-2,5-dimethoxy-benzylidene]amino]phenyl]-5-(p-tolyl)phenyl]phenyl]amine	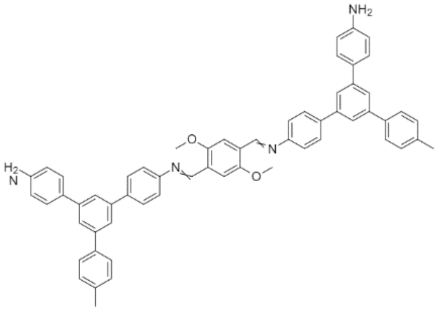	897.35771	897.35923	1.69	CID 156280204	CO	1.3048
						GD	1.3707
(1R,3R,4R,7R,9R,11S,12R,14R,16S,17R,19R,21S,22R,24R,26S,27R,29R,31S,32R,34R,35R,36R,37R,38R,39R,40R,41R,42R,43R,44R)-12,17,22,27,32-pentamethylol-2,5,8,10,13,15,18,20,23,25,28,30,33 tridecaoxaoctacyclo[29.2.2.211,14.216,19.221,24.226,29.14,9.03,7]tetratetracontane-34,35,36,37,38,39,40,41,42,43,44-undecol	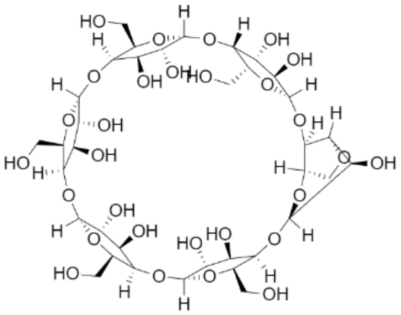	954.30609	954.30583	-0.27	CID 14056945	CO	2.7004
						GD	2.0346
						PD	2.8821
						PE	3.3357
6-aminocoumarin;6-amino-7-(2-hydroxyethyl)coumarin;6-amino-5-(2-hydroxyethyl)-4-methyl-coumarin;9-methyl-3H-pyran[3,2-e]indol-7-one;1H-pyran[2,3-f]indol-6-one	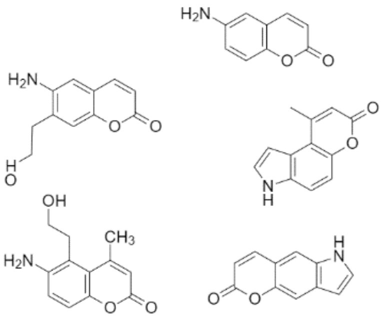	1008.28411	1008.28259	-1.5	CID 157835233	CO	2.8373
						GD	2.1350
						PD	2.7577
						PE	3.3273

$, m/z measured; &, m/z theorical; CO, Control; GD, Gestational Diabetes; PD, Pregestational Diabetes; PE, Pre-Eclampsia.

Table S14. Metabolites identified with negative ionization with ESI FT-ICR mass spectral analysis

Compound name	Candidate structure	m/z$	m/z&	Error	ID	Group	Intensity ratio
(E,E)-(E)-1,2-Diazenediylbis(N-hydroxy-1-nitromethanimine)	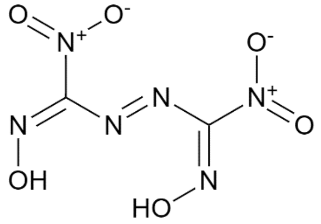	226.97841	226.97825	-0.73	67156587	CO	27.5136
						GD	197.1534
						PD	172.6607
						PE	297.8692
formic acid formyloxycarbonyloxycarbonyloxycarbonyloxycarbonyl ester	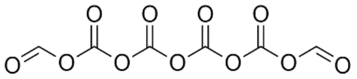	248.95442	248.95243	-7.98	CID 88027511	CO	2.3500
						GD	10.1893
						PD	11.2012
						PE	24.8862
[2-hydroxy-3-(laurylamino)propyl]-dimethyl-stearyl-ammonium	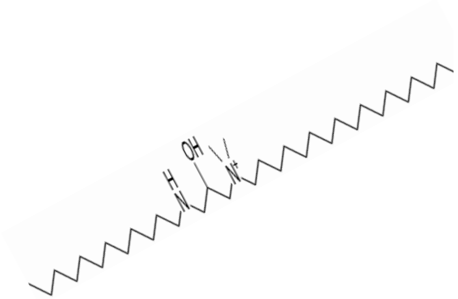	560.56105	560.56261	2.78	CID 88489499	CO	1.9101
						GD	2.1161
						PE	1.000
[3-(3,4-dimethoxy-5-propyl-phenyl)-1-methyl-2H-pyridin-5-yl]methyl-[4-[[2-(3,4-dimethoxy-5-propyl-phenyl)-4-pyridyl]methyl]cyclohexyl]-(2-methoxyphenyl)amine	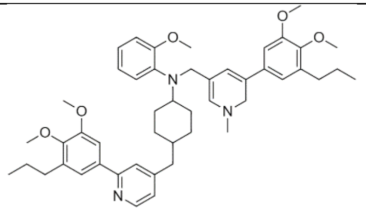	796.40905	796.41107	2.53	CID 142905999	CO	5.0747
						GD	7.0638
						PD	2.7605
						PE	1.0000

$, m/z measured; &, m/z theorical; CO, Control; GD, Gestational Diabetes; PD, Pregestational Diabetes; PE, Pre-Eclampsia.

Figures.

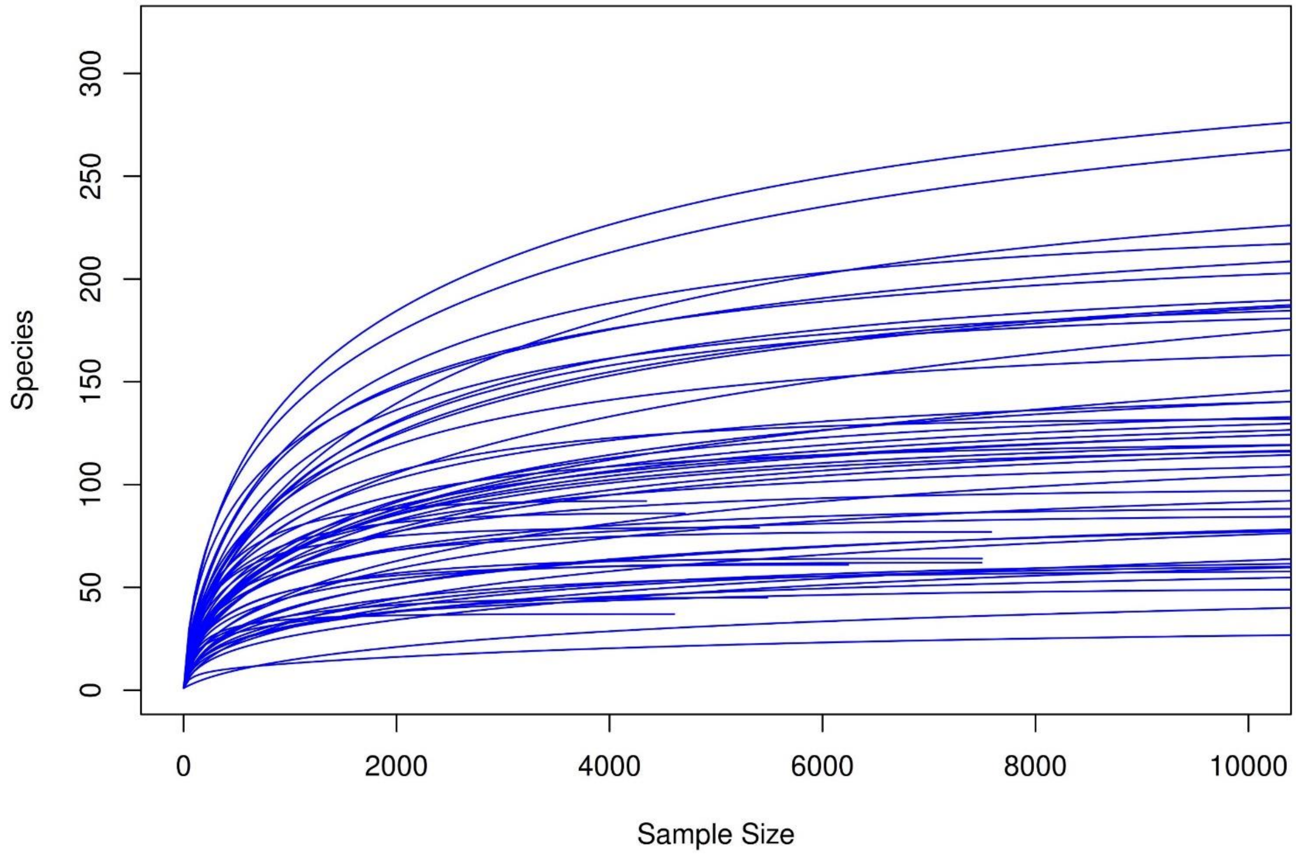

Figure S1. Rarefaction curves showing the number of bacterial species based on observed features at 97% similarity using the Silva 138 database (Accessed in July 2022). Plots show total samples for all groups in the study. Y-axis shows the number of species, and the X-axis shows the number of reads.

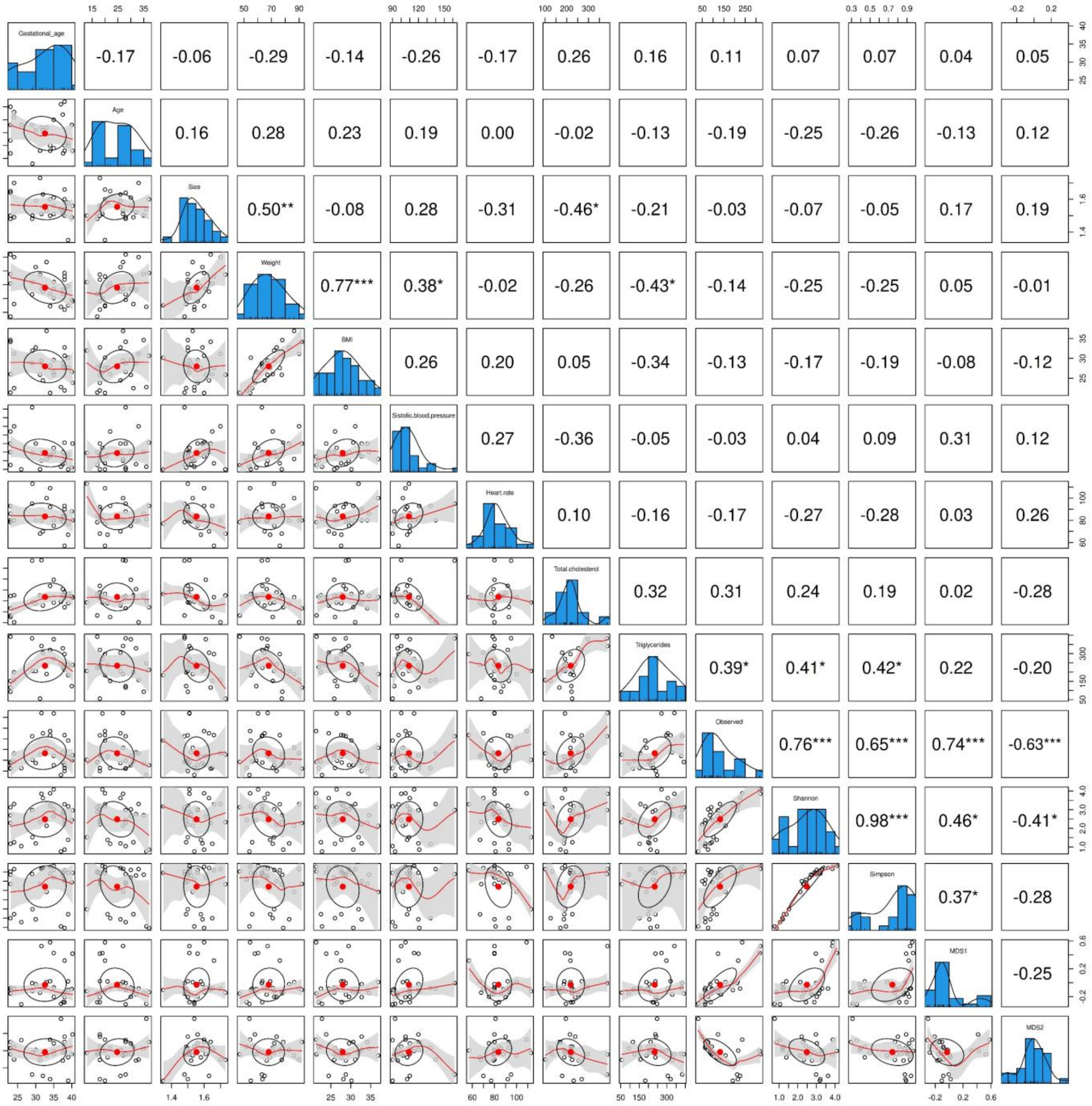

Figure S2. Correlogram showing anthropometric, biochemical and diversity data for CO group. Data correlations are indicated in the graphic squares at the left side, while numeric Spearman's rank correlation coefficients are indicated at the right side, asterisks denote $p < 0.05$ (*), $p < 0.01$ (**), $p < 0.001$ (***).

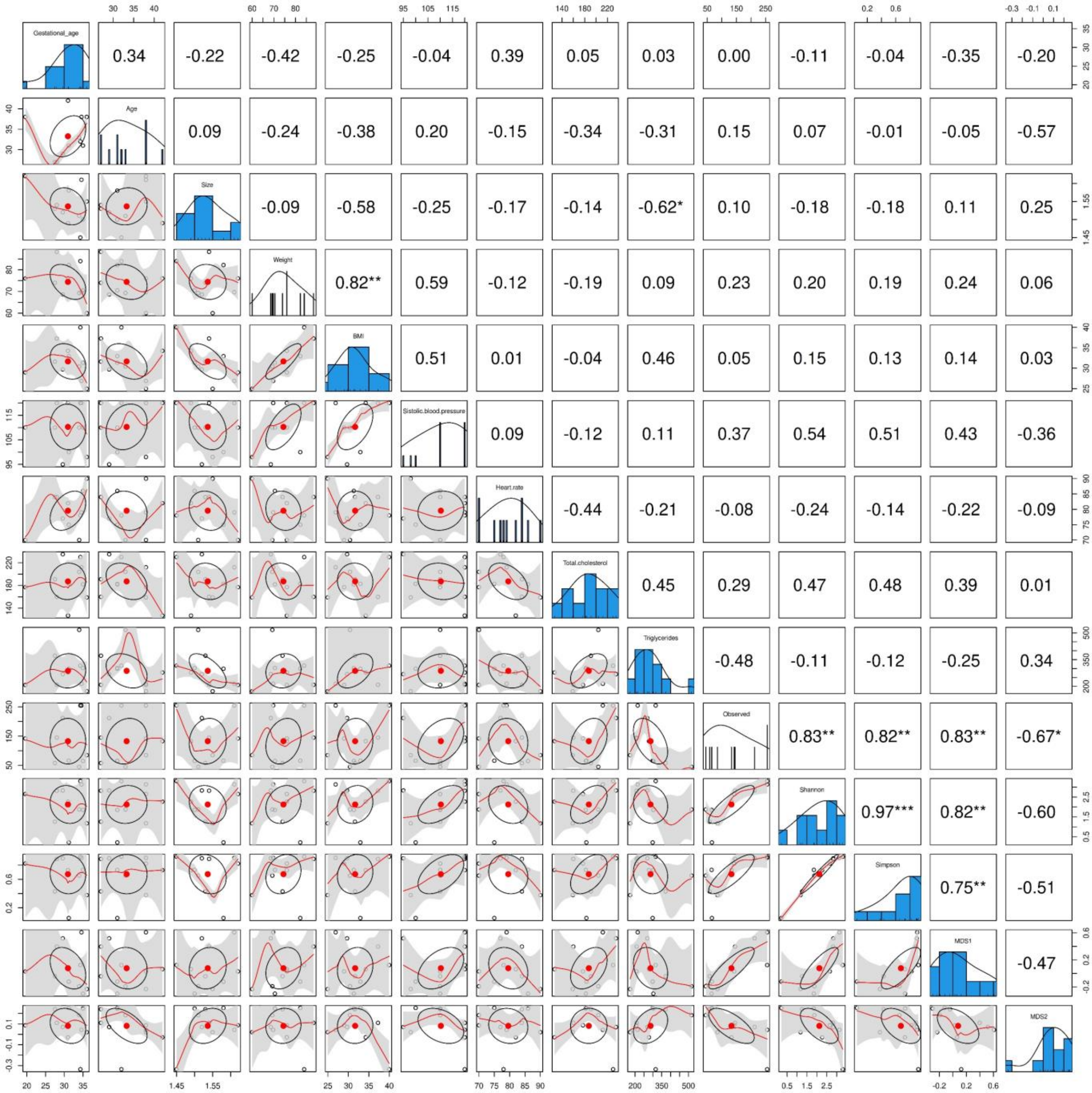

Figure S3. Correlogram showing anthropometric, biochemical and diversity data for GD group. Data correlations are indicated in the graphic squares at the left side, while numeric Spearman's rank correlation coefficients are indicated at the right side, asterisks denote $p < 0.05$ (*), $p < 0.01$ (**), $p < 0.001$ (***).

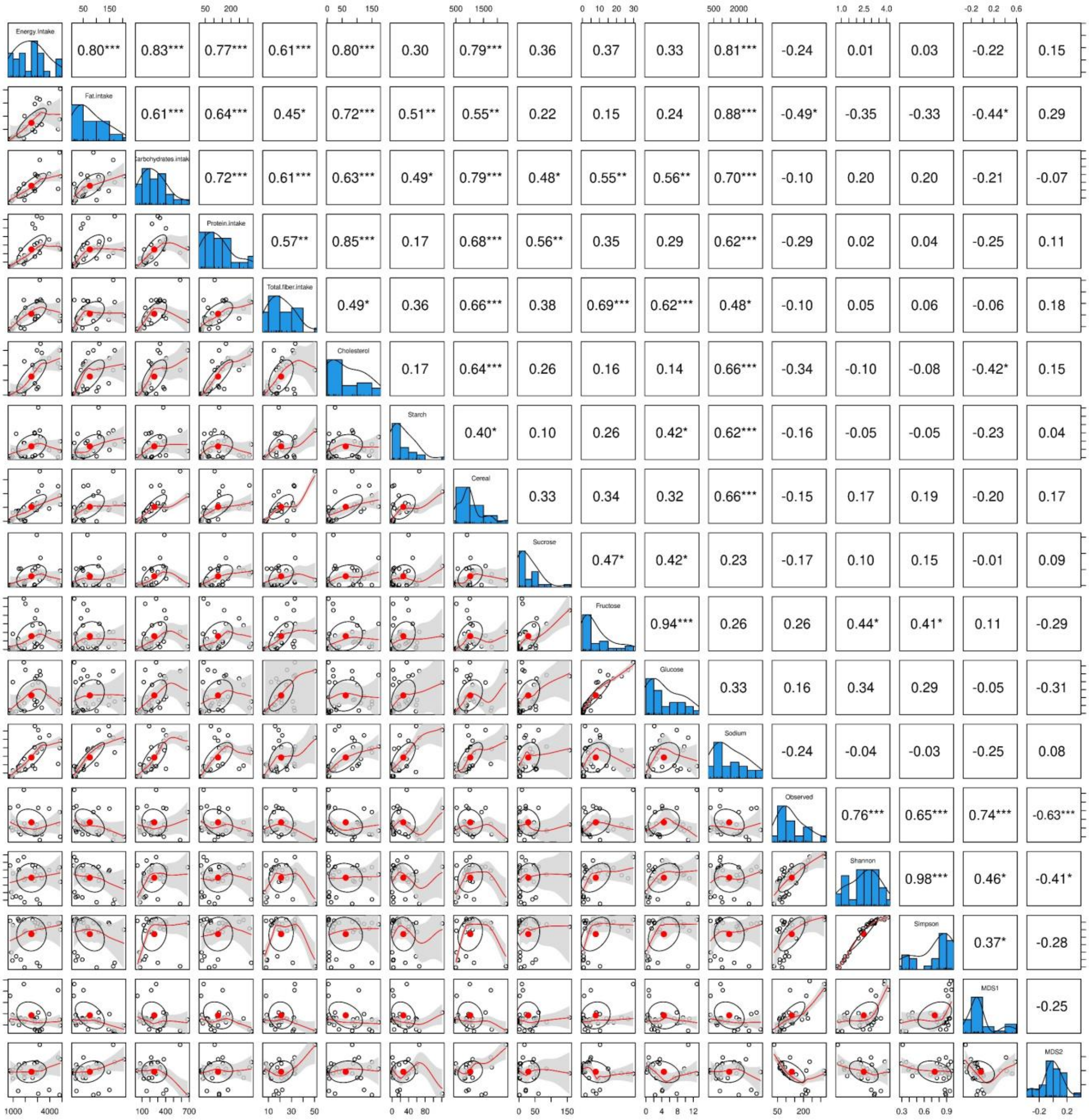

Figure S4. Correlogram showing dietary and diversity data for CO group. Data correlations are indicated in the graphic squares at the left side, while numeric Spearman's rank correlation coefficients are indicated at the right side, asterisks denote $p < 0.05$ (*), $p < 0.01$ (**), $p < 0.001$ (***).

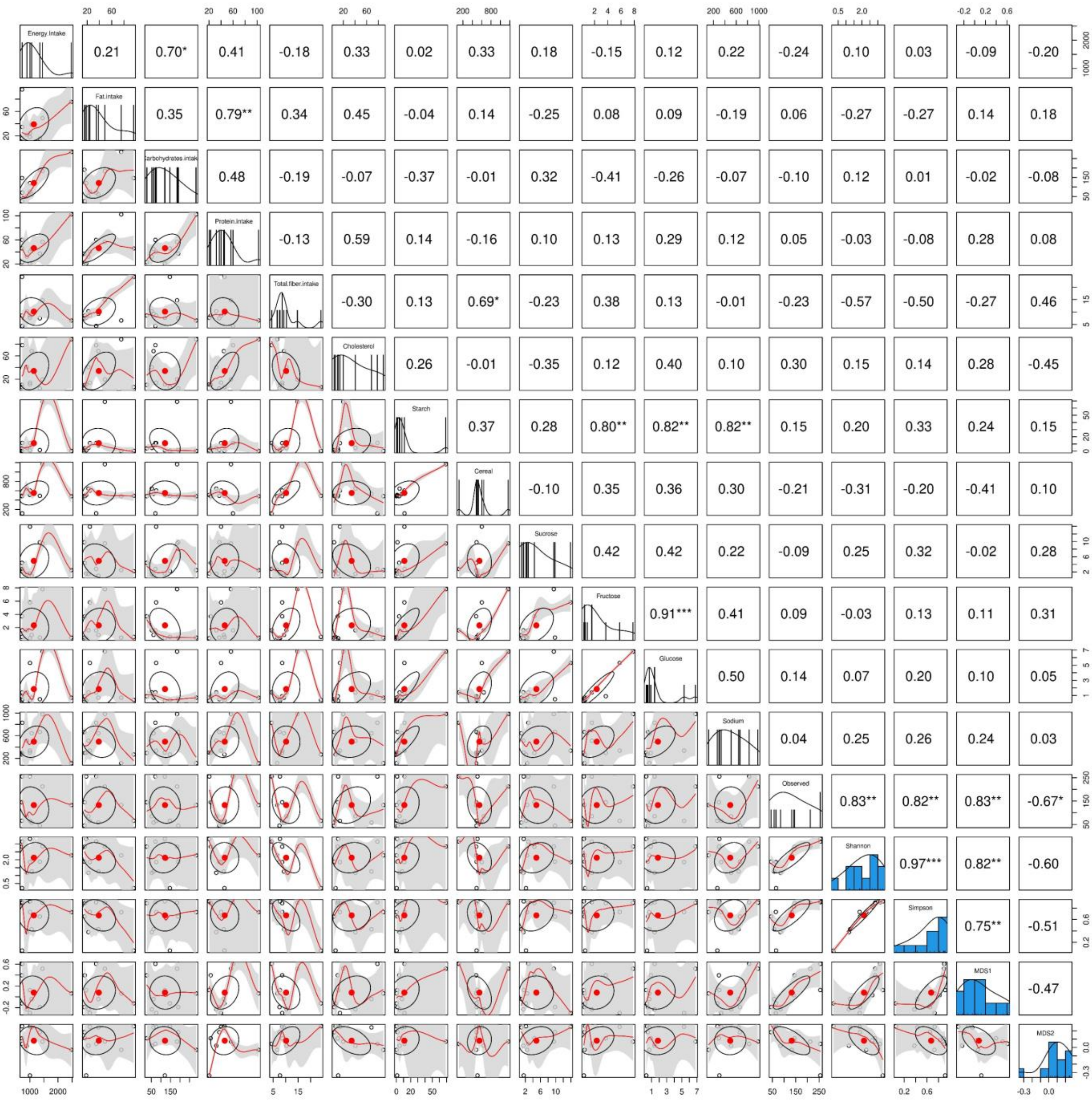

Figure S5. Correlogram showing dietary and diversity data for GD group. Data correlations are indicated in the graphic squares at the left side, while numeric Spearman's rank correlation coefficients are indicated at the right side, asterisks denote $p < 0.05$ (*), $p < 0.01$ (**), $p < 0.001$ (***).

--end-of-document--
